# Is There a Performance‐Injury Conflict Between Maximum Horizontal Deceleration and Surrogates of Noncontact Anterior Cruciate Ligament Injury?

**DOI:** 10.1002/ejsc.70014

**Published:** 2025-07-18

**Authors:** Junlei Lin, Thomas Dos'Santos, Wei Li, Xu Wang, Anthony Turner

**Affiliations:** ^1^ School of Strength and Conditioning Training Beijing Sport University Beijing China; ^2^ Force‐Velocity Laboratory Beijing Sport University Beijing China; ^3^ Department of Sport and Exercise Sciences Musculoskeletal Science and Sports Medicine Research Centre Manchester Metropolitan University Manchester UK; ^4^ Faculty of Science and Technology London Sport Institute Middlesex University London UK

**Keywords:** biomechanical determinants, deceleration, knee, noncontact ACL injury

## Abstract

This study aimed to examine the biomechanical determinants of horizontal deceleration and their correlations with noncontact ACL injury surrogates (e.g., knee joint moments). Thirty‐two male team‐sport players (Age: 21.85 ± 0.33 years; Height: 1.80 ± 0.11 m; Mass: 71.28 ± 1.39 kg) performed horizontal deceleration following 15 m sprints. Lower limb kinetics and kinematics of the first braking step were collected using 3D motion and force plates, and deceleration was assessed using radar gun. The Pearson correlation was used to determine correlations between selected variables and *p* ≤ 0.05 was considered statistically significant. Greater peak and mean horizontal deceleration were significantly correlated (*p* ≤ 0.05) with greater mean horizontal braking GRF (*r* = 0.52 and 0.41) and greater mean horizontal braking GRF ratio (*r* = 0.43 and 0.48). Greater knee joint loading (knee flexion moment, knee abduction moment, and knee internal rotation moment) were significantly correlated (*p* ≤ 0.05) with greater peak and mean vertical braking GRF (*r* = 0.30–0.41) and greater peak resultant braking GRF (*r* = 0.33–0.48). There were nonsignificant correlations between mean and peak deceleration and knee joint loading variables (*p* > 0.05). Therefore, deceleration strategies that emphasize greater horizontal and posteriorly orientated forces during the first contact of deceleration appear effective for facilitating more effective deceleration, without concomitant increases in the loading of noncontact ACL injury surrogates.

## Summary

- Horizontal decelerations with higher vertical GRFs would expose knee joint at a higher loading condition.
- Greater horizontal and posteriorly orientated forces during the first braking foot contact of deceleration appear effective for facilitating more effective deceleration.
- Athletes need to improve neuromuscular qualities (e.g., eccentric strength and reactive strength) and the ability tolerating the high braking force and knee joint loading.

## 1 Introduction

Success in most team sports is characterized by the demand of repeated high intensity acceleration and deceleration actions (Oliva‐Lozano et al. 2020). Acceleration (positive acceleration) and deceleration (negative acceleration) are highly important actions in team sports, albeit key differences in physiological and mechanical demands between both maneuvers have been observed (Hewit et al. 2011). For example, greater metabolic stress is induced during accelerations, while higher mechanical loading is observed during decelerations (Hewit et al. 2011). Moreover, a higher frequency of high‐intensity decelerations (< −3.0 m/s^2^) is performed in most team sports matches, compared to accelerations (> 3.0 m/s^2^) (Tierney et al. 2016). For example, in international rugby league, players performed high‐intensity accelerations and decelerations ∼ 20.5–28.6 times and ∼ 44.3–60.8 times, respectively, in senior teams, and ∼ 23.6–28.8 times versus 41.4–54.9 times, respectively, in the junior teams (Dempsey et al. 2018). However, despite the importance of decelerations for team sports performance as an isolated agility action, or reducing momentum prior to directional changes, greater research emphasis has been placed on understanding acceleration. Understanding the biomechanics of horizontal deceleration is a crucial component of improving this action as well as preventing lower‐limb injuries.

From a mechanical perspective, the rate of change of velocity, thus deceleration, is determined by the generation of net braking ground reaction forces (GRF) during the contact phases (thus impulse). This typically occurs over several steps, depending on the athlete's momentum (body mass and approach velocity), physical capacity, and contextual scenario. The mechanical characteristics of horizontal deceleration involve greater braking GRF and loading rates in order to reduce momentum more rapidly, compared to sprint accelerations, which have lower impact ground reaction forces and loading rates (Verheul et al. 2021). The braking function is largely associated with the horizontal component of ground reaction force (Dos'Santos et al. 2020; Dos'Santos et al. 2021a, 2021b), whereby greater and more effective braking force requires that the center of mass (COM) be positioned posterior to the center of pressure (COP, e.g., supported leg), thus attaining longer braking periods and posterior force orientation. During the braking phase, the extensors of lower limb joints serve to counteract the large external flexor moments. Thus, greater knee flexion joints moment, knee flexion angle and COM‐to‐COP distance may be correlated with greater maximal horizontal deceleration performance. However, most studies focus on deceleration performance during a COD (Dos'Santos et al. 2020; Dos'Santos et al. 2021a, 2021b), which shows large differences in kinematics and kinetics with maximal horizontal deceleration maneuvers. For example, during the COD, the athlete should reorient the COM and push off to travel into the target direction, which will influence the angles and moments of the lower limb joints. Unfortunately, limited studies have examined the fundamental mechanics required for greater maximal horizontal deceleration performance (e.g., without a COD).

Anterior cruciate ligament (ACL) injury commonly occurs during multi‐directional team sports. Notably, noncontact ACL injury has been considered as a potential career threat, as it not only requires an extensive rehabilitation period, but also induces psychological and financial stress (Kester et al. 2017). Although elaborate ACL reconstruction can be routinely performed, reduced sports‐related performance might be inevitable after return to sports (Kester et al. 2017). Notably, noncontact ACL injury, defined as an ACL tear that occurs in the absence of physical contact with other players (Ferretti et al. 1992), accounted for 44% and 88% of ACL injury mechanism in male and female football matches, respectively (Della Villa et al. 2020; Lucarno et al. 2021). Noncontact ACL injury commonly occurs during rapid and high intensity maneuvers including COD, jump‐landing, and especially during decelerations (Boden et al. 2000). Theoretically, sudden horizontal deceleration actions at high velocity impose potentially hazardous knee mechanical loading, which may exceed the tolerance capacity of the tissue, thus rupturing the ACL. Analysis reveals that noncontact ACL injury and loading of the ACL is associated with the anterior shear force generated by the quadriceps (Demorat et al. 2004), knee valgus with internal (Speer et al. 1992) or external (Fung and Zhang 2003) rotation, internal tibial rotation with near full leg extension (Arnold et al. 1979), and tibiofemoral compression loading (Meyer and Haut 2008). Specifically, both sagittal and frontal plane biomechanics are crucial determinates of ACL loading, such as lower knee flexion angle and greater posterior ground reaction force and multi‐planar knee joint loading (e.g., knee valgus and rotational moments) (Yu and Garrett 2007). Therefore, these mechanical variables have been identified as risk factors and surrogate measures of noncontact ACL injury (Yu and Garrett 2007).

Noncontact ACL injury risk is partially attributed with the techniques of various deceleration and COD maneuvers, especially techniques considered sub‐optimal (e.g., knee valgus), which impose greater mechanical loading on the knee joint, thus ACL strain. However, it is worth noting that the technical and mechanical strategies required for greater performance may also be strongly associated with higher mechanical loading, and thus predispose athletes to a higher risk of sports injuries. This paradox is known as the “performance‐injury conflict” (Dos'Santos et al. 2021a, 2021b; Fox 2018). Notably, most athletes will prefer to achieve superior performance, despite the higher mechanical loading and potential risk of injury associated with it. For example, faster COD performance was associated with higher center of mass velocity, greater braking force and knee flexion moments (KFM), but also greater knee abduction moments (KAM) (Dos'Santos et al. 2021a, 2021b). Therefore, the mechanics and techniques that contribute to faster COD performance may also be responsible for greater knee joint loading, increasing the risk of noncontact ACL injury (Dos'Santos et al. 2021a, 2021b); similar findings were also reported in previous studies (HAVENS and SIGWARD 2015; McBurnie et al. 2021; Sankey et al. 2020). However, to the best of our knowledge, it is unclear whether techniques and mechanics associated with greater horizontal deceleration without whole‐body rotation concurrently increase knee joint loading and therefore potential ACL injury risk.

Therefore, the purpose of this study was to examine the biomechanical determinants of a maximal horizontal deceleration maneuver, and their associations with surrogates of noncontact ACL injury risk (e.g., knee joint loads), similar to the approaches adopted in the COD literature (Dos'Santos et al. 2021a, 2021b). The findings of this study could improve our understanding of techniques and mechanics of maximal horizontal deceleration tasks and shed light on the performance‐injury conflict proposed within ACL injuries. It was hypothesized that mechanical characteristics associated with greater peak horizontal deceleration will concurrently increase knee joint loads, such as knee flexion moment (KFM), keen abduction moment (KAM), and knee internal rotation moment (KIRM), supporting the performance‐injury conflict during horizontal deceleration task.

## 2 Materials and Methods

### 2.1 Study Design

A cross‐sectional design was used to examine the correlations between deceleration biomechanics, horizontal deceleration performance, and noncontact ACL injury risk surrogates. All participants visited the laboratory twice, including one familiarization and one acceleration‐deceleration trials (e.g., maximal horizontal deceleration following 15 m sprint acceleration). The kinetics and kinematics of the first braking step were collected via force platform and 3D motion analysis, and velocity‐time‐distance profiles were recorded using a radar gun.

### 2.2 Subjects

A prior power analysis (G*Power V 3.1) showed that 31 participants were required for the present study. This minimal sample size was determined based on a power of 0.8, *α* level of 0.05, and correlation value of 0.42 (COD completion time and KAM) (Dos'Santos et al. 2021a, 2021b). Thirty‐two males from team sports (football: *n* = 27; rugby: *n* = 5) were recruited to take part in the study (Age: 21.85 ± 0.33 years; Height: 1.80 ± 0.11 m; Mass: 71.28 ± 1.39 kg; Back Squat relative 1RM: 1.91 ± 0.06 × body mass). Based on the participant classification framework (McKay et al. 2022), all participants were classified as trained/developmental subjects. Inclusion criteria required participants to be able to back squat ≥ 1.5x body mass, and have engaged in team sports (football, rugby, and basketball) training ≥ 2 times per week over the past 3 months, with a minimum of three years of experience in their respective sports. Participants with lower extremity injuries that may affect test results were excluded and as such, no participant had suffered a previous ACL injury. All participants were informed of the aims and procedures of this study and provided written informed consent. This study was approved by the Beijing Sport University Research Ethics Review Board for human studies.

### 2.3 Procedures

All participants performed a 20‐min standardized warm‐up session consisting of 10 min jogging and 10 min self‐selected dynamic stretching. Additionally, all participants performed three acceleration‐deceleration trials at sub‐maximal effort (80% of perceived effort). Following 5 min of passive recovery, participants performed 15 m linear sprint tests followed by maximal horizontal deceleration tests with 2–3 min of within‐test rest intervals and 5 min of between‐test rest intervals. All tests were conducted on an indoor field.

### 2.4 Linear Sprint and Maximal Horizontal Deceleration Protocols

**FIGURE 1:**
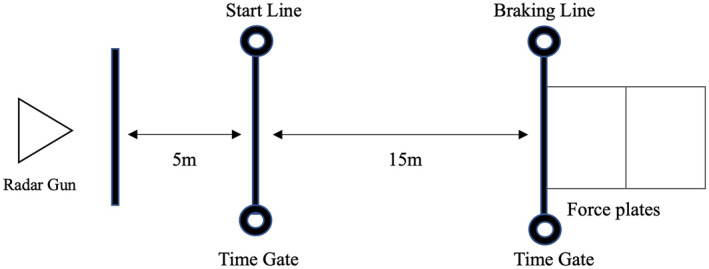
Maximal horizontal deceleration test design.

Prior to the deceleration testing, the completion time of a 15‐m linear sprint was recorded using timing gates (Smartspeed, Fusion Sport, Queensland, Australia), which were set to a height of 80 cm. To prevent a false trigger, all participants started with a two‐point staggered start position 30 cm behind the start line. Participants were instructed to sprint three trials as fast as possible. The best trial was used as a reference during maximal horizontal deceleration. Based on the maximal horizontal acceleration‐deceleration ability (ADA) test (Harper et al. 2023), a modified ADA test was used to evaluate horizontal deceleration ability in this study due to the limited lab size (Figure 1). All athletes were instructed to perform a maximal horizontal deceleration following a 15‐m linear sprint. The completion time of the 15 m sprint was recorded. To collect the velocity‐time‐distance profile, a radar device (Stalker ATS II, Applied Concepts Inc., Dallas, TX, USA) was positioned 5 m behind the start line at a height of 90 cm and sampling at 47 Hz as per manufacturer recommendations. All participants were instructed not to decelerate until passing the 15 m line, and any 15 m completion time which was 5% greater than the best 15 m sprint time recorded during the linear sprint test was discarded and subsequently another trial was performed following 2–3 min recovery, with no more than 5 additional trials. Each participant was instructed to decelerate their horizontal velocity to zero as quickly as possible during the deceleration phase, and three valid trials were recorded. The average of the three trials were used for further analysis.

Prior to the modified ADA test, reflective markers (14 mm diameter) were placed on the following bony landmarks by the lead researcher: right and left shoulder top, anterior superior iliac spine, posterior superior iliac spine, medial and lateral epicondyles of the femur, medial and lateral malleolus, the first and fifth metatarsal head, and heel of the shoe. Moreover, rigid clusters with four‐marker were secured to right and left thigh and shank.

### 2.5 Data Collection and Processing

Motion capture data and ground reaction force data of the first braking step (Figure 1) were collected using an 8‐camara set‐up (Oqus; Qualisys, Gothenburg, Sweden) at 240 Hz. Ground reaction force data were recorded from two 40 cm × 60 cm Kistler force platforms (Switzerland, Kistler 9281CA) sampling at 1200 Hz. The motion data and force data were low‐pass filtered at 15 Hz using the pipeline function of Visual 3D (C‐motion, version 6.01.12,117 Germantown, USA). Initial contact was defined as the instant when the vertical ground reaction force signal exceeded 20 N, and toe‐off was defined as the frame where the vertical ground reactive force was less than 20 N (Jones et al. 2016). The braking phase was defined as the phase from initial contact to maximal knee flexion angle. A six‐degrees‐of‐freedom model of lower limb (e.g., pelvis, thigh, shank, and foot) was developed using a static trial. A CODA pelvis orientation (Bell et al. 1989) was used to determine the location of the hip joint center and a Cardan angle sequence x‐y‐z (Grood and Suntay 1983) was used to examine the joint motion. The local coordinate system was defined at the proximal joint center for each segment. Segmental inertial properties were considered for each participants (Dempster 1955). The joint centers of knee and ankle were determined by the mid‐point of the line between lateral and medial markers. The inverse dynamic strategy (Winter 2009) was used to assess joint moment using Visual 3D.

Peak and mean deceleration performance, distance to stop, and time to stop were calculated according to a previous study (Harper et al. 2023). Kinetic and kinematic data pertaining to the first foot contact during the initial horizontal deceleration maneuver following the 15 m linear sprint were examined. For kinetic data, mean and peak vertical GRF, horizontal braking GRF (horizontal = *Anterior‐Posterior* ^2^ + *Medio‐Lateral* ^2^) (Jones et al. 2016), resultant braking GRF (resultant = *Horizontal* ^2^ + *Vertical* ^2^), and the horizontal force ratio (ratio = horizontal braking GRF/resultant braking GRF) (Jones et al. 2016) were examined.

Moreover, peak knee joint moments (sagittal, frontal, and transverse plane) were examined. Regarding kinematic data, knee angle at initial contact, peak angle, and range of motion, including multi‐planar (sagittal, frontal, and transverse plane) motion were analyzed. Furthermore, approach velocity and ground contact time were also examined based on radar data and force data, respectively.

### 2.6 Statistical Analysis

Descriptive data were presented as means ± SD. Normality was assessed using a Shapiro–Wilk test. The Pearson correlation was used to assess the correlations between horizontal deceleration performance and deceleration biomechanics, and noncontact ACL injury risk surrogates. The main assumptions were tested prior to the Pearson correlation analysis. Linear relationship between variables was assessed through scatter plots and residual plots. Potential outliers were identified through box plots and standardized z‐values (threshold set at |z| > 3) and removed where appropriate. All data met the above assumptions, thus the Pearson correlation was used in this study. The magnitude of correlations were defined as follows: trivial (0.00–0.09), small (0.10–0.29), moderate (0.30–0.49), large (0.50–0.69), very large (0.70–0.89), nearly perfect (0.90–0.99), and perfect (1.00) (Hopkins 2002). Statistical significance was defined *p* ≤ 0.05.

## 3 Results

### 3.1 Deceleration Performance Correlations

The kinetic and kinematic results of the first braking step during the deceleration task are presented in Table 1. Greater peak and mean deceleration were significantly (*p* < 0.05) and moderately‐to‐largely correlated with greater mean horizontal braking GRF (*r* = 0.41–0.52) and mean horizontal braking GRF ratio (*r* = 0.43 and 0.48) (Table 2). There were nonsignificant correlations between deceleration performance (mean and peak deceleration) and KFM, KAM, and KIRM (*r* = 0.12–0.24, *p* > 0.05) (Table S1).

**TABLE 1:** Maximal deceleration biomechanical variables descriptive statistics.

	Variable	Mean ± SD
Deceleration performance	Time to stop (s)	1.97 ± 0.35
	Distance to stop (m)	6.87 ± 1.75
	P‐deceleration (m/s^2^)	6.15 ± 0.52
	M‐deceleration (m/s^2^)	4.87 ± 0.70
	P‐approach velocity (m/s)	7.05 ± 0.86
	P‐momentum (kg·m/s)	509.37 ± 79.77
	Contact time (s)	0.14 ± 0.03
	Total braking steps	7.21 ± 1.48
Ground reaction force	P‐VGRF (BW)	3.97 ± 0.65
	P‐HGRF (BW)	−1.84 ± 0.16
	P‐RGRF (BW)	4.38 ± 0.86
	P‐HGRF‐ratio	0.43 ± 0.04
	M‐VGRF (BW)	2.34 ± 0.12
	M‐HGRF (BW)	−0.96 ± 0.18
	M‐RGRF (BW)	2.54 ± 0.16
	M‐HGRF‐ratio	0.38 ± 0.06
Joint moment (risk factors)	P‐KFM (Nm/kg)	4.13 ± 1.27
	P‐KAM (Nm/kg)	1.22 ± 1.14
	P‐KIRM (Nm/kg)	−0.78 ± 0.25
	P‐HFM (Nm/kg)	−4.07 ± 1.34
	P‐ADFM (Nm/kg)	−1.45 ± 0.34
Knee joint angle (risk factors)	P‐KFA (˚)	−92.97 ± 7.36
	P‐KAA (˚)	−6.98 ± 3.31
	P‐KIRA (˚)	−2.15 ± 4.06
	IC‐KFA (˚)	−35.36 ± 7.39
	IC‐KAA (˚)	1.01 ± 1.66
	IC‐KIRA (˚)	−1.21 ± 3.84
	ROM‐KFA (˚)	57.61 ± 8.68
	ROM‐KAA (˚)	7.99 ± 1.63
	ROM‐KIRA (˚)	1.04 ± 1.64

Abbreviations: ADFM, ankle dorsi‐flexion moment; HFM, hip flexion moment; HGRF, horizontal ground reaction force; HGRF‐Ratio, horizontal ground reaction force ratio; IC, initial contact; KAA, knee abduction angle; KAM, knee abduction moment; KFA, knee flexion angle; KFM, knee flexion moment; KIRA, knee internal rotation angle; KIRM, knee internal rotation moment; M, mean; P, peak; RGRF, resultant ground reaction force; ROM, range of motion; VGRF, vertical ground reaction force.

**TABLE 2:** Correlation values for variables with significant associations with maximal horizontal deceleration performance.

	Variable	*r*	*p*
P‐deceleration	M‐HGRF	0.52	0.017
	M‐HGRF‐ratio	0.43	0.002
M‐deceleration	M‐HGRF	0.41	0.021
	M‐HGRF‐ratio	0.48	0.012

Abbreviations: HGRF, horizontal ground reaction force; HGRF‐Ratio, horizontal ground reaction force ratio; M, mean; P, peak; *r,* Pearson correlation coefficient.

### 3.2 Knee Joint Loading Correlations

Greater peak KFM was significantly (*p* < 0.05) and moderately correlated with shorter ground contact time (*r* = −0.36), higher peak resultant (*r* = 0.48) and vertical (*r* = 0.32) braking GRF, higher mean resultant (*r* = 0.34) and vertical (*r* = 0.30) braking GRF. Greater peak KFM was significantly correlated with higher KAM (*r* = 0.52) and KIRM (*r* = 0.69), the strength of correlation was large (Table 3).

**TABLE 3:** Correlation values for variables with significant associations with multi‐planar knee joint loading.

	Variable		*r*	*p*
KFM	Contact time		−0.36	0.029
	Knee joint moment	P‐KAM	0.52	< 0.001
		P‐KIRM	0.69	< 0.001
	RGRF	P‐RGRF	0.48	0.039
		M‐RGRF	0.34	0.036
	P‐GRF	Vertical component	0.32	0.024
		Horizontal component	0.18	0.411
	M‐GRF	Vertical component	0.30	0.003
		Horizontal component	0.17	0.390
KAM	Knee joint moment	P‐KIRM	0.79	< 0.001
	RGRF	P‐RGRF	0.33	< 0.001
		M‐RGRF	0.42	0.013
	P‐GRF	Vertical component	0.38	< 0.001
		Horizontal component	0.24	0.283
	M‐GRF	Vertical component	0.31	0.021
		Horizontal component	0.22	0.296
KIRM	Contact time		−0.45	0.018
	RGRF	P‐RGRF	0.41	< 0.001
		M‐RGRF	0.48	0.006
	P‐GRF	Vertical component	0.36	< 0.001
		Horizontal component	0.24	0.272
	M‐GRF	Vertical component	0.41	0.004
		Horizontal component	0.30	0.113

Abbreviations: KAM, knee abduction moment; KFM, knee flexion moment; KIRM, knee internal rotation moment; M, mean; P, peak; *r,* Pearson correlation coefficient; RGRF, resultant ground reaction force; VGRF, vertical ground reaction force.

Greater peak KAM was significantly correlated (*p* < 0.05) with higher peak resultant braking GRF (*r* = 0.33), higher vertical braking GRF (*r* = 0.38), higher mean resultant braking GRF (*r* = 0.42), higher mean vertical braking GRF (*r* = 0.31). Greater peak KAM was also significantly (*p* < 0.05) and largely correlated with higher peak KRM (*r* = 0.79), the strength of correlation was very large (Table 3).

Greater peak KIRM was significantly correlated (*p* < 0.05) with shorter ground contact time (*r* = −0.45), higher peak resultant braking GRF (*r* = 0.41), higher peak vertical braking GRF (*r* = 0.36), higher mean resultant braking GRF (*r* = 0.48), higher mean vertical braking GRF (*r* = 0.41). The magnitude of these relationships was moderate (Table 3).

## 4 Discussion

The aim of this study was to examine the biomechanical determinants of maximal horizontal deceleration, and their correlations with noncontact ACL injury risk surrogates. The results of this study found that greater deceleration performance (peak and mean deceleration) was significantly correlated with greater mean anterior‐posterior braking GRF, greater horizontal braking GRF and greater horizontal braking GRF ratio (Table 2). Nonsignificant correlations were observed between (mean and peak) deceleration and peak approach velocity, peak approach momentum, KAM, KFM, and KIRM (*p* > 0.05). Therefore, deceleration strategies that emphasize greater horizontal and posteriorly orientated forces during the first braking foot contact of deceleration appear effective for facilitating more effective deceleration, without concomitant increases in the loading of noncontact ACL injury surrogates.

### 4.1 Correlations With Horizontal Deceleration

Horizontal deceleration is characterized by a reduction in forward speed. Thus, similar to sprint acceleration, horizontally orientated GRF has been identified as ‘effective force’ (MORIN et al. 2011). The greater horizontal component of braking GRF contributed to greater horizontal deceleration performance. In the present study, we found that greater (peak and mean) deceleration performance was significantly and moderately‐to‐largely correlated with greater mean horizontal force and greater mean horizontal force ratio during the first initial braking foot contact (Table 2). This finding is in line with previous studies (Dos'Santos et al. 2020), where the authors reported that greater horizontal braking GRF was required to achieve a greater horizontal deceleration capability during a COD task. Notably, the ability to apply horizontally orientated braking GRF is not only dependent on muscular qualities, but also on technique. For example, the greater COM‐to‐COP distance, the greater ratio of horizontal to vertical braking GRF magnitude, thus affecting the GRF vector direction.

### 4.2 Correlations With Knee Joint Loading

The essence of horizontal deceleration is underpinned by rapid dissipation/transfer of energy (negative work) through the generation of eccentric force of the lower limb (hip, knee, and ankle) during the weight acceptance phase, thereby reducing the approach momentum. In the sagittal plane, we found that the knee joint displayed the greatest range of motion (∼57.61°) (Table 1), indicating a “knee‐dominant” strategy in horizontal deceleration maneuvers. This finding suggested that the knee joint displays a crucial role in energy dissipation during horizontal deceleration. Therefore, it is unsurprising that greater resultant braking GRF is required to be counteracted by greater eccentric lengthening of knee extensors (KFM), thus increasing strain on the ACL and imposing larger shear force on the knee joint (HAVENS and SIGWARD 2015). Moreover, greater KFM was largely and very largely correlated with greater KAM and KRM, respectively (Table 3), indicating that KAM and KRM were increased in line with increases in resultant braking GRF. These findings are in accordance with a previous study that demonstrated significant correlations between resultant braking GRF and KRM (peak braking GRF: *ρ* = −0.458, mean braking GRF: *ρ* = −0.505, *p* ≤ 0.001) and KAM (peak braking GRF: *ρ* = 0.306, mean braking GRF: *ρ* = 0.488, *p* ≤ 0.001) during final braking step of 90° cutting tasks, which involves deceleration with whole‐body rotation (Dos'Santos et al., 2021a, 2021b). Whether the deceleration task involves full‐body rotation or not, the keen joint loading is largely determined by the magnitude of braking GRF. Therefore, the greater vertical component of peak and mean braking GRF was largely responsible for the greater overall knee joint loading (e.g., KAM, KFM, and KRM), whereas greater horizontal braking GRF ratio is responsible for greater horizontal performance.

Interestingly, we did not find that greater deceleration performance correlated with higher risk of noncontact ACL injury surrogates (KFM, KAM, and KRM), or with any variables that also correlated with higher knee loading (Tables 2 and 3). This unexpected finding does not support our hypothesis regarding the “performance‐injury conflict” in horizontal deceleration without whole‐body rotation maneuvers. Notably, this finding does not suggest there is no risk of noncontact ACL injury surrogates during horizontal deceleration tasks without whole‐body rotation. Because of the substantial knee joint loading observed during maximal horizontal deceleration, there is a potential for increased strain on the ACL, which may elevate the risk of injury. During the horizontal deceleration maneuver, the higher knee loading was correlated with larger braking GRF (Table 3), but not with the faster approach velocity and momentum. Unlike the deceleration with whole‐body rotation of COD, whereby the author reported that greater KRM was largely associated with faster approach velocity and thus greater braking GRF (Dos'Santos et al. 2021a, 2021b). These findings suggested that the whole‐body rotation during deceleration would be a risk factor of ACL injury. Therefore, these variables were significantly related with higher horizontal deceleration performance (deceleration without whole‐body rotation), but not with noncontact ACL injury surrogates, whereas higher overall knee loading was observed during the deceleration task.

### 4.3 Injury Risk Factors of Horizontal Deceleration

It is important to note that, as mentioned above, noncontact ACL injuries are largely determined by various factors, including high braking forces, shear forces and compressive forces etc. Thus, increases in any of these factors has the potential to expose the knee joint to a greater loading context. Notably, high knee joint loading is one of the key factors in increased ACL loading and therefore a higher risk of noncontact ACL injury. The biomechanical characteristics of horizontal deceleration maneuvers involve high loading rates and high impact force applied over a short contact duration (Lozano‐Berges et al. 2021; Verheul et al. 2021, 2019), which exposes the knee joint to greater loading conditions. We found that the peak braking GRF on average amounted up to 4.38 times body mass (peak VGRF: 3.97 times body mass, peak HGRF: 1.84 times body mass) at the first braking foot contact, and it even reached up to 6 times body mass in a previous study (Verheul et al. 2021). The braking forces during horizontal deceleration are larger than that of high intensity COD tasks. Dos’Santos et al. (2021a, 2021b) investigated the biomechnical determinants of the 90° cutting task (the distance between starting line and the position of the final foot contact is 15 m), and they reported that the peak resultant braking force was approximately equal to 3 times their body mass at the penultimate and final foot contact. Moreover, the peak resultant braking forces for the penultimate and final foot contact in both the traditional and modified 505 tasks (15 m acceleration distance) were relatively low, about 2.6–2.8 times their body mass (Dos'Santos et al. 2020). Moreover, we found that greater peak braking GRF was moderately associated with greater overall knee joint loading (Table 2), supporting that greater braking force induces greater knee joint loading. Therefore, high braking forces in horizontal tasks are one of the important risk factors of noncontact ACL injuries.

Furthermore, performing high‐intensity braking tasks with shallow knee flexion angles is also one of the key injury risk factors of noncontact ACL injuries. Most cases of noncontact ACL injury occurred with the knee in near full extension, for example, deceleration with whole‐body rotation of COD, and landing from a jump and pivoting with the knee in full extension (Bahr and Krosshaug 2005; Boden et al. 2000). Cadaveric studies found greater ACL loading at 0°‐ 45° of knee flexion angle, with 30° of knee flexion angle recognized as a high‐risk position (Withrow et al. 2006); ACL loading was not as high at knee flexions > 60° (Arms et al. 1984). This is because a shallow flexion angle increases anterior tibial shear, which increases the ACL loading. We noted that the knee flexion angle was 35.36° at initial contact of the first step in the horizontal deceleration tasks. Although our study did not reveal a statistically significant relationship between knee joint loading and knee flexion angle at initial contact, it is important to note that shallow knee flexion angles are widely recognized in the literature as a biomechanical risk factor for increased ACL loading.

## 5 Limitations

Only the lower limbs were examined in the present study. However, the motion of the trunk also affects the kinetic and kinematics of the lower limbs and may also influence the overall loading of the knee joint. Furthermore, the findings of the present study may only be applicable to the population we investigated. It requires further investigations to extrapolate these findings to other populations, sports, and genders, including those returning from ACL reconstruction. Finally, we only examined the first braking step and thus representative of the early braking steps. Future research should consider multiple foot contact to better understand the performance and injury risk biomechanical determinants.

## 6 Practical Applications

The findings of the present study showed that greater maximal deceleration performance (peak and mean deceleration) was associated with the greater anterior‐posterior braking force and the greater ratio of horizontally oriented braking GRF. Therefore, practitioners should develop players' technique in maximally and effectively applying greater horizonal braking GRF. For example, practitioners could coach players to adopt a position in which the center of mass should be positioned posterior to the center of pressure (e.g., supported leg). Moreover, athletes need to improve neuromuscular qualities (e.g., eccentric strength and reactive strength) and the ability tolerating the high braking force and knee joint loading. These recommendations aimed to reduce risk of ACL injury by developing physical capacity and neuromuscular control without compromising the maximal deceleration performance.

## 7 Conclusion

To the best of our knowledge, this is the first to examine the biomechanical determinants of a maximal horizontal deceleration maneuver, and their associations with surrogates of noncontact ACL injury risk. As such, deceleration strategies which emphasize greater horizontal and posteriorly orientated forces during the first braking foot contact of deceleration appear effective for facilitating more effective deceleration, without concomitant increases in the loading of noncontact ACL injury surrogates.

## Conflicts of Interest

The authors declare no conflicts of interest.

## Supporting information

Table S1
