# Supplementary material for: Is There a Performance‐Injury Conflict Between Maximum Horizontal Deceleration and Surrogates of Noncontact Anterior Cruciate Ligament Injury?
Source: Eur J Sport Sci. 2025 Jul 18;25(8):e70014. doi: 10.1002/ejsc.70014 (PMC12273744; doi:10.1002/ejsc.70014)
Supplement: Supplementary file 1 — Table S1 [file EJSC-25-e70014-s001.docx]

Supplemental Table 1. Correlations between maximal deceleration performance and multi-

planar knee joint loading.

|  | **Variable** | ***r*** | ***p*** |
| --- | --- | --- | --- |
| M-Deceleration | P-KFM | 0.12 | 0.231 |
|  | P-KAM | 0.17 | 0.374 |
|  | P-KIRM | 0.21 | 0.832 |
| P-Deceleration | P-KFM | 0.16 | 0.266 |
|  | P-KAM | 0.22 | 0.673 |
|  | P-KIRM | 0.24 | 0.128 |

Note: P peak, M mean, KFM knee flexion moment, KAM knee abduction moment, KIRM knee internal rotation moment, *r* Pearson correlation coefficient
